# Unconscious Male With Reported Seizure

**DOI:** 10.1016/j.acepjo.2025.100136

**Published:** 2025-04-11

**Authors:** Naleen Patel, Bradley S. Jackson, Nima Sarani

**Affiliations:** Department of Emergency Medicine, University of Kansas Medical Center, Kansas City, Kansas, USA

**Keywords:** POCUS, point-of-care ultrasound, aortic dissection, subxiphoid

## 1 Case Presentation

**Figure 1:**
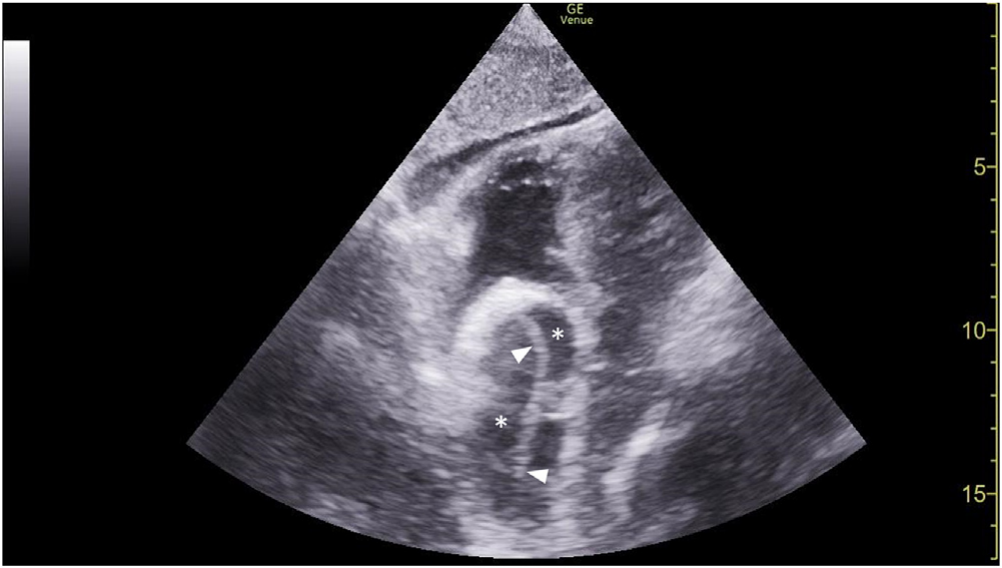
Subxiphoid point-of-care ultrasound showing ascending aorta (asterisks) with an echogenic linear dissection flap (arrowheads).

**Figure 2:**
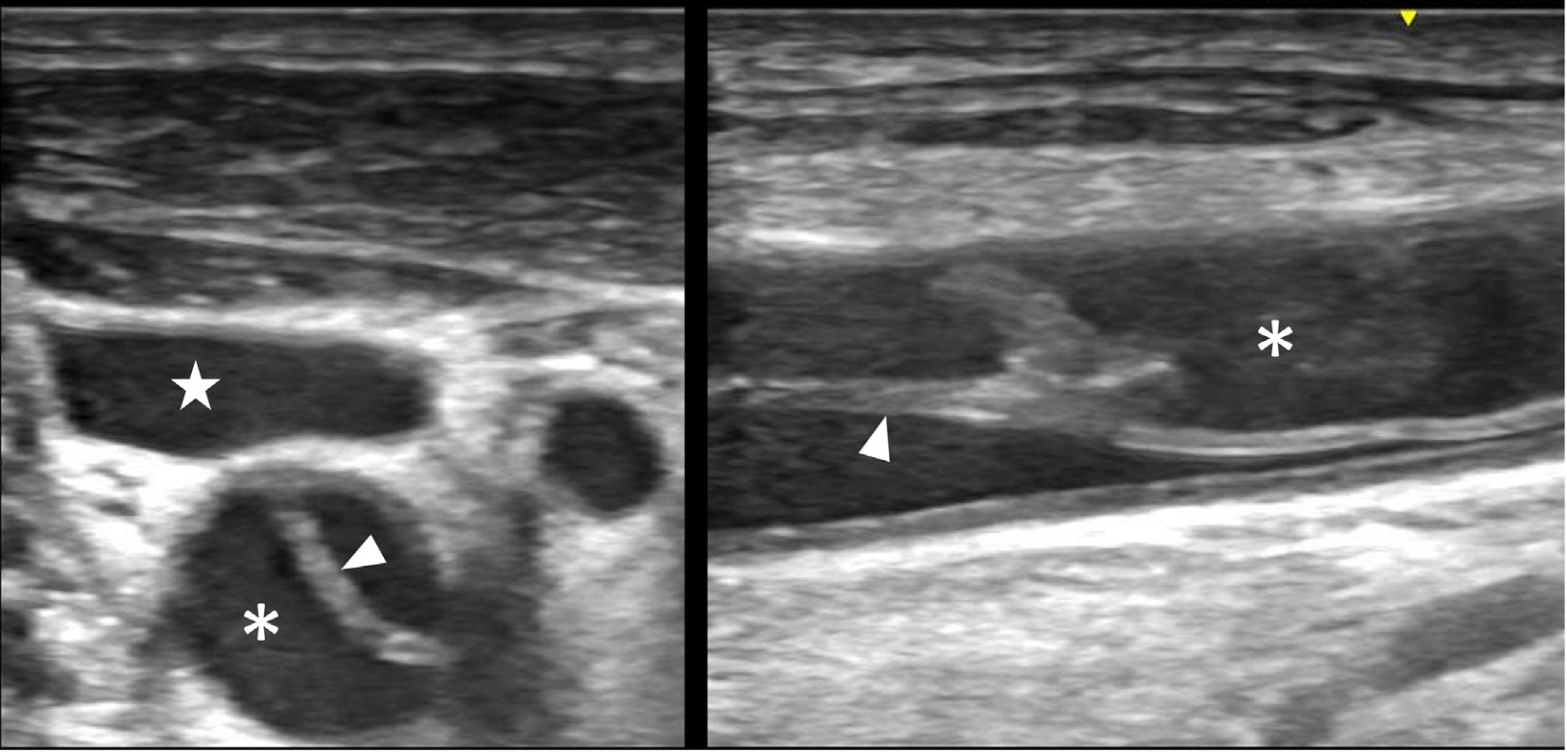
Transverse-axis (left panel) and long-axis (right panel) views of point-of-care right neck ultrasound demonstrating an internal jugular vein (star), carotid artery (asterisk), and echogenic linear carotid dissection flap (arrowhead).

An unidentified 68-year-old male with an unknown medical history was brought to the emergency department via ambulance after bystanders witnessed the patient suffering seizure-like activity at the library. Intramuscular midazolam was administered prior to arrival. He presented with agonal breathing requiring bag ventilation with a Glasgow Coma Scale score of 3. He was intubated for airway protection. Prior to transport for head imaging, he had a transient episode of postintubation hypotension that prompted a cardiac point-of-care ultrasound examination (Fig 1, Video 1). These findings prompted bedside carotid ultrasound (Fig 2, Video 2).

**Video 1:**
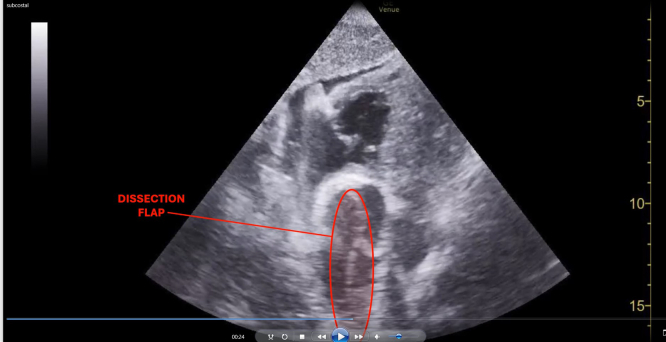
Subxiphoid point-of-care ultrasound showing ascending aorta with a dissection flap.

**Video 2:**
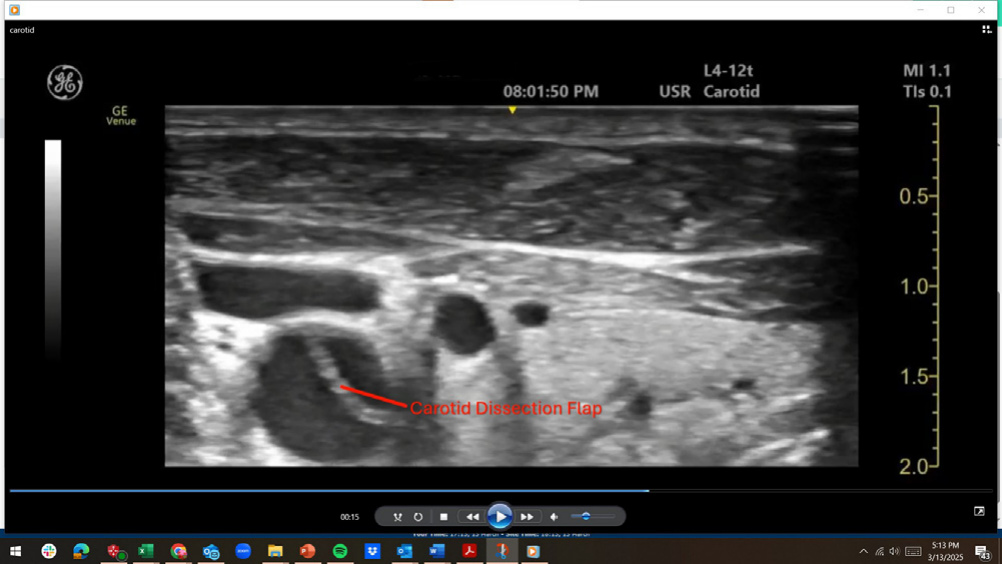
Transverse-axis point-of-care ultrasound of the right neck demonstrating carotid dissection.

## 2 Diagnosis: Stanford Type A Dissection With Bilateral Carotid Extension

**Figure 3:**
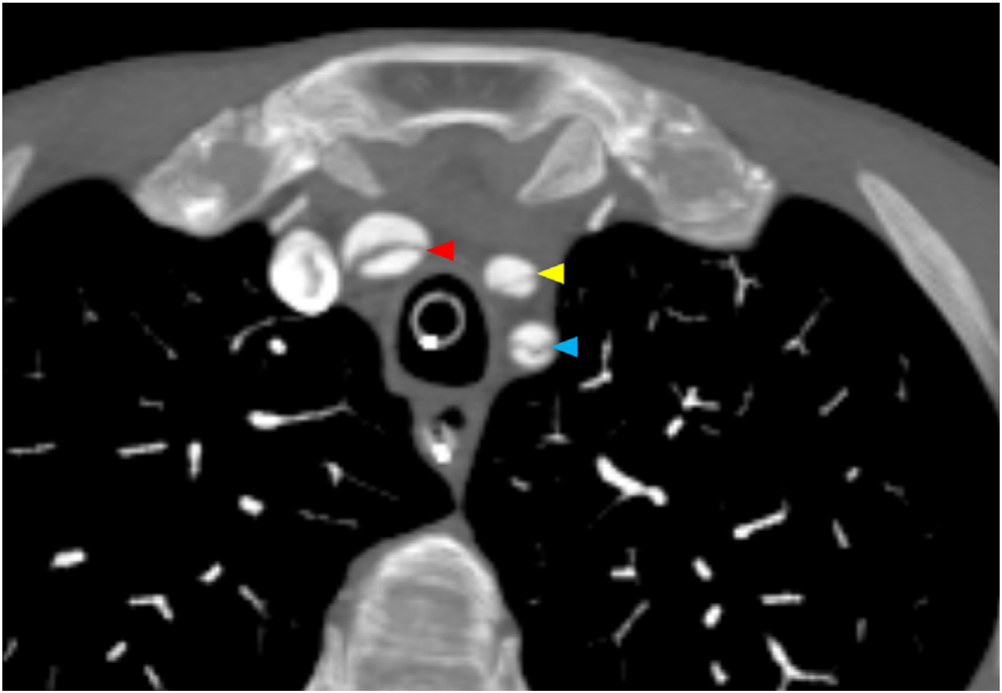
Transverse computed tomography angiography of the neck showing dissection flaps (arrowheads) in the brachiocephalic (red), left common carotid (yellow), and left subclavian (blue) arteries.

**Figure 4:**
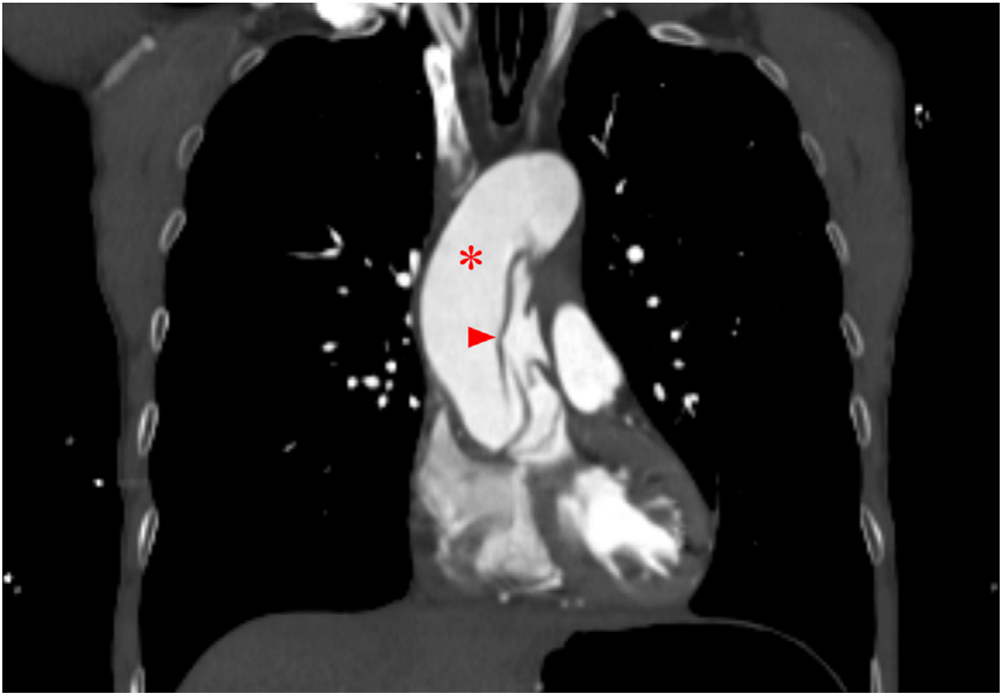
Coronal computed tomography angiography of the chest showing an ascending aorta (asterisk) with a dissection flap (arrowhead).

The subxiphoid view noted a prominent aortic root with direct visualization of a dissection flap. This prompted esmolol initiation and computed tomography angiography imaging of the torso, head, and neck. Stanford type A dissection was confirmed with extension into all branches of the aortic arch, most critically into the left carotid artery with M1 involvement (Figs 3 and 4, Video 3).

**Video 3:**
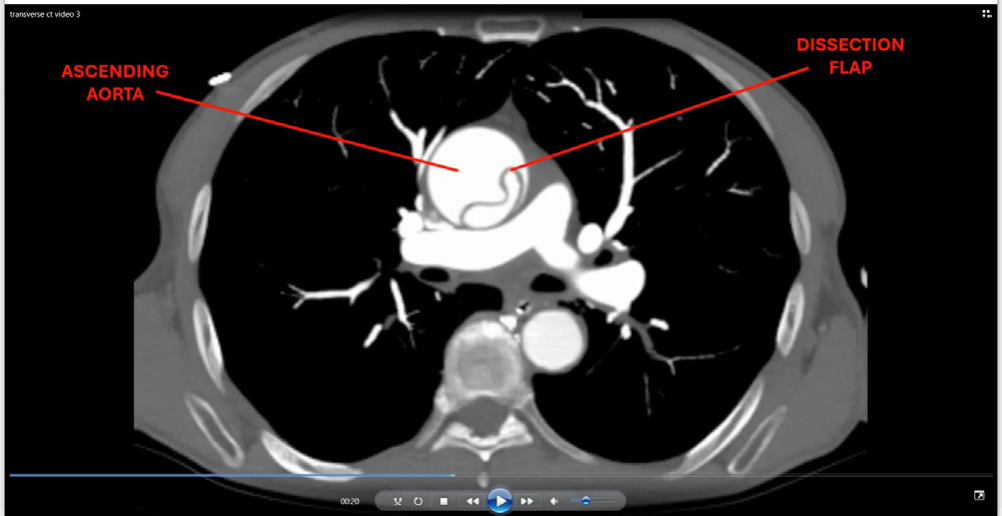
Transverse-axis computed tomography angiography showing a dissection flap in the ascending aorta with extension into brachiocephalic, left common carotid, and left subclavian arteries.

Blaivas et al^1^ previously described the subxiphoid view as capable of indirectly suggesting proximal thoracic aortic dissection when the Mercedes-Benz sign was noted, highlighting this window’s potential utility when conventional imaging is delayed or unavailable. Here, direct subxiphoid visualization of the dissection flap was used for diagnosis, which was especially useful as the patient’s other cardiac windows were uninterpretable.

Cardiothoracic surgery was emergently consulted prior to computed tomography angiography performance, given point-of-care ultrasound findings. Ultimately, given the extent of the dissection and his poor prognosis, surgical intervention was felt not feasible. The patient unfortunately passed away days later.

## Funding and Support

The author(s) received no financial support for the research, authorship, and/or publication of this article.

## Conflict of Interest

All authors have affirmed they have no conflicts of interest to declare.
